# The Effect of Training on Erythrocyte Energy Status and Plasma Purine Metabolites in Athletes

**DOI:** 10.3390/metabo10010005

**Published:** 2019-12-19

**Authors:** Barbara Pospieszna, Krzysztof Kusy, Ewa Maria Słomińska, Wioleta Dudzinska, Monika Ciekot-Sołtysiak, Jacek Zieliński

**Affiliations:** 1 Department of Athletics, Strength and Conditioning, Poznan University of Physical Education, Królowej Jadwigi 27/39, 61-871 Poznan, Poland; kusy@awf.poznan.pl (K.K.); ciekot@awf.poznan.pl (M.C.-S.); 2 Department of Biochemistry, Medical University of Gdansk, Dębinki 1, 80-211 Gdansk, Poland; eslom@gumed.edu.pl; 3 Department of Physiology, Faculty of Biology, The University of Szczecin, ul. Felczaka 3c, 71-412 Szczecin, Poland; wiola@univ.szczecin.pl

**Keywords:** adenylate metabolites, purine derivatives, HGPRT, training periodization

## Abstract

This study aimed to assess the changes in red blood cell (RBC) energy status and plasma purine metabolites concentration over a one-year training cycle in endurance-trained (EN; *n* = 11, 20–26 years), and sprint-trained (SP; *n* = 11, 20–30 years) competitive athletes in comparison to recreationally-trained individuals (RE; *n* = 11, 20–26 years). Somatic, physiological, and biochemical variables were measured in four training phases differing in exercise load profile: transition, general, specific, and competition. Significantly highest values of RBC adenylate energy charge (AEC; *p* ≤ 0.001), ATP-to-ADP and ADP-to-AMP ratios (*p* ≤ 0.05), and plasma levels of adenosine (Ado; *p* ≤ 0.05) were noted in the competition phase in the EN and SP, but not in the RE group. Significantly lowest plasma levels of adenosine diphosphate (ADP; *p* ≤ 0.05), adenosine monophosphate (AMP; *p* ≤ 0.001), inosine (Ino; *p* ≤ 0.001), and hypoxanthine (Hx; *p* ≤ 0.001) accompanied by higher erythrocyte hypoxanthine-guanine phosphoribosyltransferase (HGPRT) activity (*p* ≤ 0.001), were observed in the competition phase in both athletic groups. No significant alterations were found in the erythrocyte concentration of guanine nucleotides in any group. In conclusion, periodized training of competitive athletes’ results in a favorable adaptation of RBC metabolism. The observed changes cover improved RBC energy status (increased AEC and ATP/ADP ratio) and reduced purine loss with more efficient erythrocyte purine pool recovery (increased HGPRT activity and plasma levels of Ado; decreased Hx and Ino concentration).

## 1. Introduction

Mammalian erythrocyte (RBC), a unique cell without a nucleus, serves as a model cell in many studies. Apart from its main functions, such as carrying oxygen and carbon dioxide, and controlling the acid-base balance level, erythrocytes also exert other roles. They take part in nitric oxide (NO) metabolism, influence blood rheological properties, and exert the erythrocrine function by releasing vast amounts of ATP and other bioactive molecules [1,2]. All these properties are essential for human response to exercise, especially that of maximal intensity when substantial homeostasis disturbances occur [2,3,4]. Repeated high-intensity exercise leads to profound homeostasis disturbances, such as local hypoxia, acidification, hypoglycemia, or hyperthermia and, thus, to increased post-exercise hemolysis and, consequently, to increased erythropoiesis during restitution [5,6]. Therefore, in athletes, compared to sedentary humans, besides increased erythropoiesis, there is also observed enhanced erythrocyte turnover and an increase in the number of young red blood cells [2,3]. 

The RBCs’ vitality, resilience, and functioning rely on their energy metabolism, mainly glycolysis, which is the only source of adenosine triphosphate (ATP) in RBC [7]. Since ATP resynthesis involves multistep metabolic pathways, the amount of RBC energy resources is usually described by the concentration of ATP, adenosine triphosphate/adenosine diphosphate ratio (ATP/ADP), and the adenylate energy charge (AEC) [8]. 

There are scant studies reporting changes in purine nucleotide catabolite concentration in human and animal erythrocytes in response to single bouts of exercise [3,9,10,11]. The results obtained from humans suggest that both maximum- [3,9] and moderate-intensity [10] exercise do not significantly affect the total adenylate and guanylate concentration, while adenine (but not guanine) nucleotide concentration in the adenylate pool considerably changes. A substantial post-exercise decrease in adenosine diphosphate (ADP) and adenosine monophosphate (AMP) concentration leads to an increase in ATP/ADP ratio, ADP/AMP ratio, and AEC. These observations are in line with data derived from animals [11]. 

The research conducted so far demonstrated that there were significant differences between trained and sedentary individuals in erythrocyte energetics (the ATP/ADP and ADP/AMP balance shifted towards ATP and ADP, increasing AEC) [3], which may be indicative of higher metabolic activity, more effective ATP synthesis, and better response of erythrocytes to exercise-induced increases in energy demand [3]. However, there is no data concerning the differences in red blood cell metabolism between recreationally trained and highly-trained individuals (competitive athletes), whose training regimes differ essentially in both time frames and exercise loads. Precise annual periodization is the key feature distinguishing high-performance from recreational training. In the latter case, exercise loads usually do not change throughout the year or are reduced according to individual engagement in physical practice. Periodization ensures that athletes achieve their best possible sports performance during their most important competition. For this reason, a one-year training plan of highly-trained athletes is divided into specifically designed training phases (preparation: general and specific, competition and transition), which considerably differ in training goals and structure. The training loads (exercise intensity, volume, and frequency of sessions) change throughout consecutive training phases to reach the maximum intensity and low volume in the competition phase [12]. In disciplines based on running, sprint and endurance training regimes have usually equal time frames but very different characteristics of training loads resulting from distinct training goals. 

Thus far, changes in erythrocyte energy status and purine nucleotide catabolites concentration were not yet explored also during a longer period or under prolonged training regime. Reports based on one-year training cycles only focused on changes in plasma hypoxanthine (Hx) concentration and erythrocyte hypoxanthine-guanine phosphoribosyltransferase (HGPRT) activity [13,14,15]. It was demonstrated that sprint training, predominated by high-intensity exercise, but also endurance training supplemented by high-intensity loads, led to a decrease in plasma Hx and an increase in erythrocyte HGPRT activity. The magnitude of the changes depended on the alterations in the quantity of high-intensity "anaerobic" exercise training loads. Training cessation resulted in an increase in post-exercise Hx concentration and a decrease in erythrocyte HGPRT activity [16,17]. 

The research on the link between erythrocyte energetics and whole-body functioning, e.g., during various physical efforts, may give new insight into some aspects of exercise and training physiology. We expect that the indicators of RBC energy status and the level of ATP metabolites are sensitive to changes in training specificity and exercise loads and, thus, they could be used as a valuable tool indicating the training status in athletes. 

Thus, this study aimed to assess whether the change in training loads during a periodized one-year cycle is associated with the changes in erythrocyte energy status and plasma purine metabolites concentration in highly-trained athletes. We hypothesized that the erythrocyte energy status will adapt to the alterations in training loads throughout the phases of the annual training cycle. 

## 2. Results

### 2.1. Somatic and Exercise Characteristics

The somatic and exercise characteristics of studied participants in consecutive phases of the annual training cycle are shown in Table 1. Significant differences (*p* ≤ 0.001) between consecutive phases of the annual training cycle were observed in the EN group for weight, body mass index (BMI), and maximal oxygen uptake (VO_2max_), while in the SP group for weight, BMI, VO_2max_, Hct, and lactate (LA) at rest.

During the studied training cycle, only weight and Hb changed significantly in the RE group (*p* ≤ 0.05). In all study examinations, there were significant differences between analyzed groups in weight, BMI, Hb concentration, Hct, and VO_2max_ (*p*-values from <0.001 to 0.025). LA concentration at rest was significantly different between groups in the general and competition phases (*p* ≤ 0.05).

### 2.2. Erythrocyte Adenine Nucleotides

**Figure 1:**
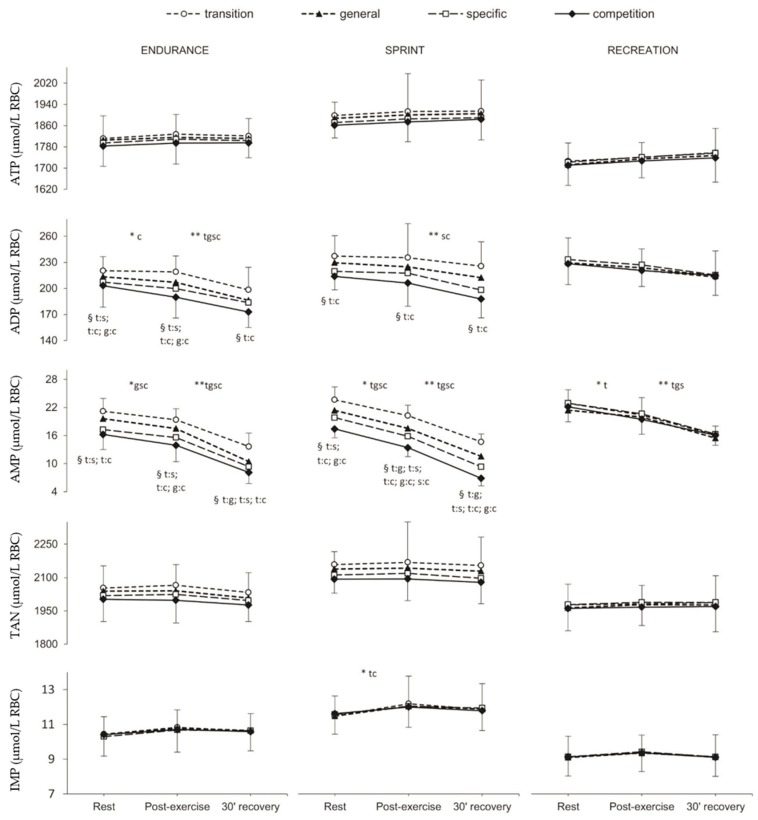
Red blood cell adenine nucleotides (ATP, ADP, AMP), total adenine nucleotide (TAN) and inosine nucleotide (IMP) concentration at rest, post-exercise and after 30 min of recovery in endurance runners (*n* = 11), sprinters (*n* = 11) and recreational runners (*n* = 11) in consecutive training phases (transition, general, specific, competition). Values are given as means ± SD. ATP: adenosine-5’-triphosphate; ADP: adenosine-5’-diphosphate; AMP: adenosine-5’-monophosphate; TAN: total adenine nucleotides; IMP: Inosine-5’-monophosphate. TAN (total adenine nucleotides) = [ATP] + [ADP] + [AMP]. Training phases: t: transition, g: general, s: specific, c: competition. * Significant difference between rest and post-exercise. ** Significant differences between Post-exercise and 30’ recovery. § Significant differences between training phases (e.g., t:s indicates the difference between transition and specific phase).

Figure 1 presents the changes in red blood cell adenine nucleotides, TAN, and inosine nucleotides at rest, post-exercise and during the recovery over the annual training cycle. Statistically significant changes in ADP and AMP between the consecutive training phases of the annual training cycle were only noticeable in athletic groups (EN and SP) (*p* ≤ 0.05). These changes occurred mainly between transition and specific and between transition and competition phases, as measured at rest, immediately post-exercise, and after 30 min of recovery. The differences between measurements at rest, post-exercise and recovery were observed in endurance athletes (ADP and AMP), sprinters (ADP, AMP, and inosinomonophosphate (IMP)) and in the recreationally-trained group (only AMP) (Figure 1; *p* ≤ 0.05). 

Endurance athletes differed significantly from sprinters only in ATP concentration in the transition, specific, and competition phases of the annual training cycle. There were significant differences between sprinters and recreational runners in the level of ATP (*p* ≤ 0.001), TAN (*p* ≤ 0.05), and IMP (*p* ≤ 0.001) in all examinations, and in AMP concentration (*p* ≤ 0.05) in the specific and competition phases. The differences between EN and RE groups were significant for ATP in the transition and specific phases (*p* ≤ 0.001), for ADP and AMP in the specific and competition phases (*p*-values ranged from <0.001 to 0.036), and for IMP concentration (*p* ≤ 0.001) in the general, specific, and competition phases of the annual training cycle. 

### 2.3. Erythrocyte Energy Status

**Figure 2:**
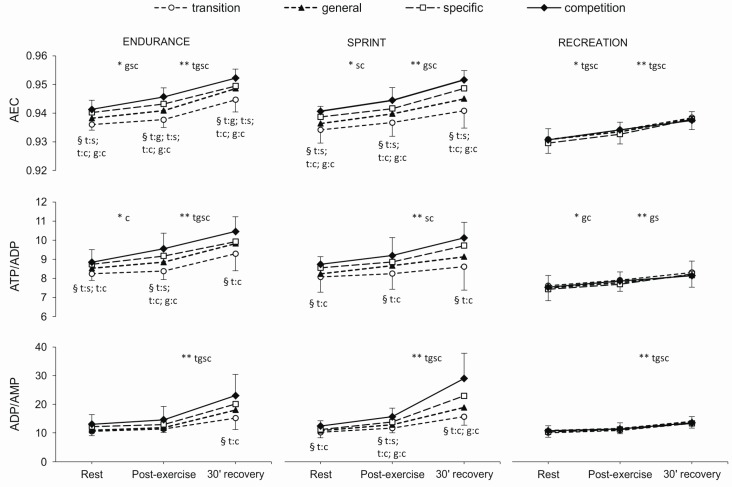
Red blood cell energy status (AEC, ATP/ADP, ADP/AMP) at rest, post-exercise and after 30 min of recovery in endurance runners (*n* = 11), sprinters (*n* = 11) and recreational runners (*n* = 11) in consecutive training phases (transition, general, specific, competition). Values are given as means ± SD. AEC: adenylate energy charge; ATP/ADP: adenosine-5’-triphosphate/adenosine-5’-diphosphate ratio; ADP/AMP: adenosine-5’-diphosphate/ adenosine-5’-monophosphate ratio. AEC (adenylate energy charge) was evaluated according to the formula by Atkinson AEC = ([ATP] + 0.5[ADP]) / ([ATP] + [ADP] + [AMP]). Training phases: t: transition, g: general, s: specific, c: competition. * Significant difference between rest and post-exercise. ** Significant differences between post-exercise and 30’ recovery. § Significant differences between training phases (e.g., t:s indicates the difference between transition and specific phase).

As indicated in Figure 2, changes in AEC and ATP/ADP and ADP/AMP ratios observed throughout the consecutive training phases were significant only in highly-trained athletes (EN and SP). In both sprint and endurance groups, the differences between measurements at rest, post-exercise, and after 30 min of recovery were observed mainly in the specific and competition phases (*p*-values ranged from <0.001 to 0.025). Significant differences were also noted in the RE group (*p*-values ranged from <0.001 to 0.005). 

There were no significant differences between the EN and SP groups. Following significant between-group differences occurred in the level of AEC and ATP/ADP ratio (*p*-values ranged from <0.001 to 0.004): (i) EN vs. RE group (AEC in all phases, ATP/ADP in general, specific, and competition phases), and (ii) SP vs. RE group (both variables in all phases except transition). 

### 2.4. Erythrocyte HGPRT Activity

**Figure 3:**
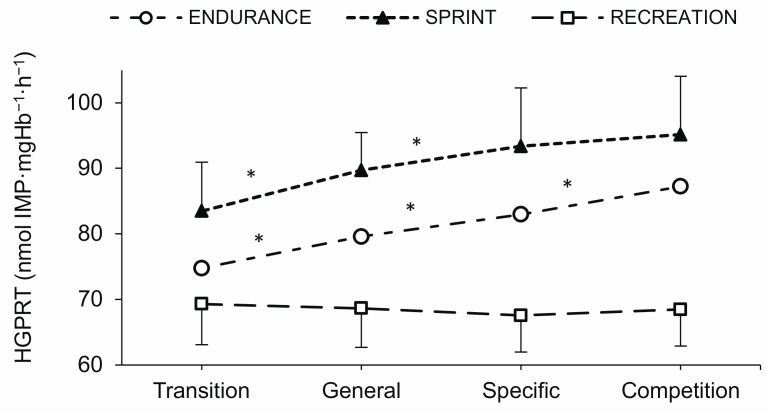
Red blood cell hypoxanthine-guanine phosphoribosyltransferase (HGPRT) activity in endurance runners (*n* = 11), sprinters (*n* = 11), and recreational runners (*n* = 11) in consecutive training phases (transition, general, specific, competition). * significantly different from previous training phase, *p* ≤ 0.001.

The between-group differences in resting erythrocyte HGPRT activity in consecutive training phases are shown in Figure 3. In the EN group, there were significant differences between all consecutive study examinations (*p* ≤ 0.001). In the SP group, the differences were observed between the transition and general and between the general and specific training phases (*p* ≤ 0.001). There were no differences in the level of erythrocyte HGPRT activity in the annual training cycle in the RE group. There were significant differences in erythrocyte HGPRT activity between all analyzed groups in each training phase (*p* ≤ 0.001). 

### 2.5. Plasma Purine Nucleotide

**Figure 4:**
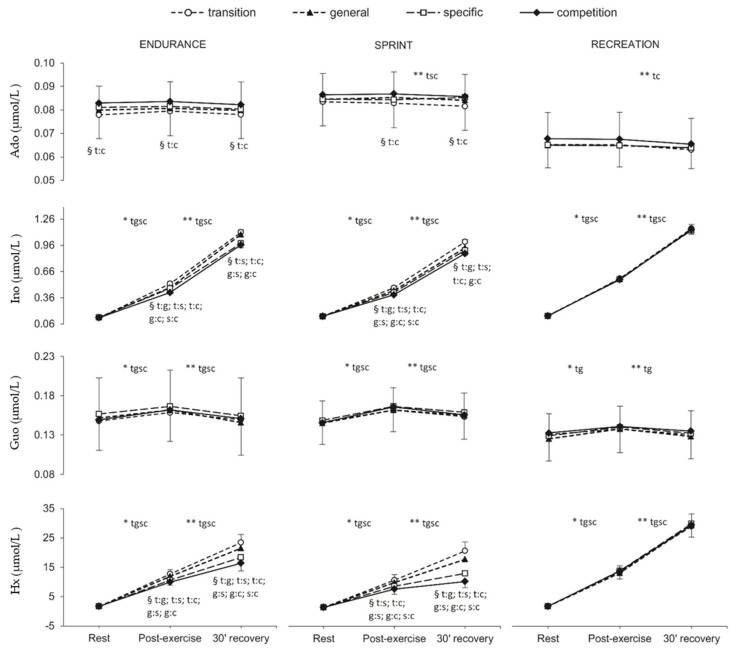
Plasma adenosine (Ado), inosine (Ino), guanosine (Guo), and hypoxanthine (Hx) concentration at rest, post-exercise, and after 30 min of recovery in endurance runners (*n* = 11), sprinters (*n* = 11) and recreational runners (*n* = 11) in consecutive training phases (transition, general, specific, competition).Values are given as means ± SD. Training phases: t: transition, g: general, s: specific, c: competition. * Significant difference between rest and post-exercise. ** Significant differences between post-exercise and 30 min recovery. § Significant differences between training phases (e.g., t:s indicates the difference between transition and specific phase).

Figure 4 shows the changes in plasma Ado, Ino, Guo, and Hx concentrations in consecutive study examinations and subsequent measurement points during each exercise test in all three studied groups. The most pronounced differences were noted in EN and SP groups for Ino (*p* ≤ 0.001) and Hx (*p* ≤ 0.001) concentrations measured immediately after exercise and after 30 min of recovery. There were also significant changes in Ado concentration between the transition and competition phases in the EN group in all three measurement points and in the SP group immediately after exercise and 30 min of recovery. Significant changes between rest and post-exercise values as well as between post-exercise and recovery values were observed in all measurement points (*p* ≤ 0.001) in the EN and SP groups. In the RE group, significant changes were shown for Ino, Hx (all measurement points s, *p* ≤ 0.001), and Guo (only in the transition and general phases, *p* ≤ 0.023). The concentration of Ado changed significantly only between post-exercise and 30-min recovery values in the SP (transition, specific, and competition phases, *p* ≤ 0.001) and in the RE group (transition and competition phases, *p* ≤ 0.038). 

There were significant differences in plasma Hx concentration between all analyzed groups in each training phase (*p* ≤ 0.001). Significant differences (*p* ≤ 0.002) occurred between SP and RE groups in the level of Ado in all training phases and between EN athletes and RE group in Ino concentration in the competition phase of the annual training cycle (*p* ≤ 0.002). 

### 2.6. Erythrocyte Guanine Nucleotides

In general, no significant differences were found in the erythrocyte concentration of most guanine nucleotides, as assessed at rest, post-exercise, and after 30 min of recovery in four consecutive phases of the annual training cycle in any group and between measurement points during the progressive test (Table S1).

## 3. Discussion

To our best knowledge, this is the first study to analyze the changes in adenine nucleotide concentrations and the RBC energy status in a longer training cycle. We demonstrated that the specialized one-year training, of both sprint- and endurance-type, resulted in a favorable adaptation of the erythrocyte energy status in competitive athletes. Significant increments in erythrocyte AEC and ATP/ADP ratio were shown from the transition to the competition phase. Admittedly, the concentration of guanine nucleotides did not change throughout the annual training cycle in any studied group, but it was consistently higher in athletes comparing to the recreationally-trained runners. Similarly to previous studies [13,14,15], we showed that erythrocyte HGPRT activity peaked in the competition phase. Moreover, a gradual reduction in plasma Ino and Hx concentrations and increased plasma Ado concentration were visible in competitive athletes from the transition to the competition training phase. 

### 3.1. Erythrocyte Energy Status 

#### 3.1.1. Adenylate and Energy Status

The available evidence concerning the changes in purine nucleotide catabolites concentration in erythrocyte in response to the single physical effort is scarce. Research conducted so far included animals [11] and both sedentary [9] and trained [3,10] humans. The decline in erythrocyte ADP and AMP after a moderate to high-intensity exercise has been previously described by Yamamoto, et al. [10] and Dudzinska, et al. [3,9]. As our research demonstrates, ADP and AMP decline is also observed after exercise until exhaustion. Basically, AMP concentration should decrease due to the deamination reaction (AMP to IMP). Our data, however, exclude such possibility because we did not observe an increase in IMP concentration and a concurrent decrease in TAN and AEC. Knowing that the concentration of ATP and ADP is always determined by the rate of phosphorylation and dephosphorylation reactions, our results indicate that propelled phosphorylation reactions (ADP to ATP and AMP to ADP) are responsible for the increase in ATP/ADP and ADP/AMP ratios and AEC. We showed that the training-induced adaptive changes are associated with the changes in the adenine nucleotide concentration in the adenylate pool and their ratios, rather than with an increase in the total adenylate concentration. The observed increase in AEC and ATP/ADP ratio in the competition phase indicates a higher erythrocyte potential for ATP-dependent processes to meet energy needs, leading in consequence to better exercise tolerance [7].

Although the pattern and direction of changes in the RBC energy status due to the effect of exercise were similar between the studied groups, the differences between competitive athletes and recreationally-trained runners became visible throughout the annual training cycle. Significantly higher ATP/ADP and ADP/AMP ratios, as well as AEC value across consecutive training phases (from transition to competition) in highly-trained athletes, indicate that the adaptation to high-intensity training loads plays a key role. Such an adaptation signifies a more effective ATP production and probably an increased activity of anabolic reactions dependent on ATP inflow, including repair mechanisms. Thus, enhancing the RBC energy potential may be considered as one of the links in the process of adaptation to increased tolerance to high-intensity exercise.

Results obtained in our study suggest that the longitudinal changes observed in athletes are mainly related to an increase in the energy potential of the RBC and the enzymatic activity in the purine resynthesis pathway. The differences in erythrocyte energy status between athletes and controls are most likely due to training-induced increments in RBC turnover, resulting in shorter RBC lifespan and a higher proportion of younger RBCs in trained individuals [2,3,5,6]. 

#### 3.1.2. Erythrocyte Guanine Nucleotides

Guaninetriphosphate (GTP) plays a key role in protein synthesis, it is a substrate in the cyclic guaninemonophosphate (GMP) synthesis and a potential source of energy. However, due to the significantly lower concentration in the erythrocyte, guanine purines exert a smaller impact on erythrocyte bioenergetics compared to adenine [7,18].

In our study, in line with previous ones [3,10], the concentrations of guanine nucleotides did not change after a single exercise test. Additionally, we revealed that these concentrations also remained unchanged throughout the annual training cycle in all studied groups. Therefore, one can assume that the concentrations of erythrocyte guanine nucleotides do not reflect the exercise response or training adaptation and cannot be considered as adequate biomarkers of the RBC energy status.

### 3.2. Erythrocyte HGPRT Activity

We found that in the control group the activity of erythrocyte HGPRT, an enzyme involved in RBC purine resynthesis, did not change throughout the investigated training period and that it remained significantly lower compared to the athletes. A similar difference was previously shown between sedentary people and trained rowers [3]. In our earlier studies, we showed that erythrocyte HGPRT activity increased during an annual training cycle in both young [13,14] and middle-aged [15] competitive athletes. Similarly, in this current research, the erythrocyte HGPRT activity was lowest in the transition and highest in the competition phase. This indicates an increasingly effective purine recovery in the erythrocyte through the *salvage* pathway. Other researchers observed similar responses in the skeletal muscle after a strenuous short-term exercise [19,20]. It implies that the training-related enhancement of the purine resynthesis mechanism has a systemic effect.

### 3.3. Plasma Purine Derivatives

In highly-trained athletes, but not in the control group, the increased amount of intense training loads in the subsequent phases of the annual training cycle resulted in adaptive changes in plasma purine derivatives concentration. The significantly lowest levels of plasma Ino and Hx in the competition phase indicate either their reduced release from the skeletal muscle [20,21] or their more effective reutilization to IMP and further incorporation to the adenylate pool through a purine nucleotide cycle (not present in RBC) [22]. Our observation that high-intensity training brings about reduced purine loss is consistent with earlier findings. Following a 1- and 4-day high-intensity interval training (HIIT), a decreased urinary Hx concentration was shown in young active men, signifying a training-induced adaptation in purine nucleotide metabolic pathways [21]. Stathis, et al. [20] observed that in recreationally active individuals the skeletal muscle concentration of Ino was reduced after a 30-second sprint performance test after a seven-week sprint training, which indicated a reduction of exercise purine loss. 

An important phenomenon we observed was the significantly higher Ado concentration in endurance and sprint-trained athletes in the competition compared to the transition phase. Because such changes were not found in the recreationally-trained group, it seems that the inclusion of high-intensity workouts, including frequent hypoxia episodes, leads to an increase in Ado production, which source in hypoxia is the extracellular AMP hydrolysis catalyzed by ecto-5’-nucleotidase [23]. This assumption is supported by the reports on the effects of swimming training in rats. Langfort, et al. [24] observed an increase in the heart 5’-nucleotidase activity after endurance and sprint training. Roque, et al. [25] showed enhanced extracellular adenine nucleotides hydrolysis, increased activity and expression of heart 5’-nucleotidase, and increased coronary blood flow and angiogenesis. This suggests that Ado exerts a strong effect on the adaptation to exercise by affecting the angiogenesis regulation, erythropoietin production, plasma norepinephrine, and epinephrine levels, myocardial glucose uptake, and alternating blood flow, glycogenolysis, systolic blood pressure, and respiration [26,27,28].

The strength of this study is the inclusion of two homogenous groups of top-level athletes from national teams, trained by the same coaches. Training loads were precisely scheduled and their volume and intensity changed according to the aim of each training phase. To our best knowledge, this is the first study to comprehensively analyze purine metabolism (13 purine nucleotides) in a longer period in highly-trained athletes of two distinct specializations.

There are also some limitations. Elite athletes of different specializations have different genetic predispositions and are subject to sports selection at an early age. In future research, it would be interesting to analyze to what extent the observed changes and differences in RBC energy status and purine metabolism relate to genetic factors. It would be also beneficial to rate the activity of glycolytic enzymes, which is the only metabolic pathway that produces ATP in the human red blood cell. The conversion of intracellular AMP and ADP into ATP requires two reactions, i.e., the phosphorylation of AMP to ADP by adenylate kinase and the phosphorylation of ADP to ATP by pyruvate kinase and phosphoglycerate kinase. Hence, it is possible that long-term training can lead to specific adaptive changes in the activity of enzymes in the ATP-generating pathway. The combined assessment of enzyme activity, especially regulatory enzymes, the blood concentration of purine nucleotides, and their metabolites would allow for a better assessment of RBC energy metabolism and a deeper insight into the mechanisms behind the observed differences. The limiting aspect of this study may also be the lack of precise control of dietary intake in the recreationally-trained group. We have familiarized them with standard nutritional recommendations for physically active people, however, we did not precisely control to what extent they adhered to them.

## 4. Materials and Methods 

### 4.1. Subjects

The study included three groups of male adult athletes, two of which comprised endurance- and sprint-trained athletes from the Polish national teams, who competed at the national and international level. The endurance-trained group (EN; *n* = 11) included triathletes, aged 22.9 ± 2.12 (20–26) years, practicing competitive sport for 8.27 ± 1.62 (6–11) years, specializing in the standard Olympic distance (1.5 km swimming, 40 km cycling, 10 km running). The second athletic group included sprint-trained men (SP; *n* = 11), aged 23.55 ± 3.27 (20–30) years, practicing competitive sport for 8.18 ± 0.98 (7–10) years, specializing in the 100 m, 200 m, and 4 × 100 m relay races. The control group consisted of recreationally-trained runners (RE; *n* = 11), aged 22.73 ± 2.15 (20–26) years, competing in half marathons, marathons, or road/cross-country running, but not subjected to a periodized training regimen. Over the research period, there were no significant differences between the studied groups as regards age (*p* = 0.737), body height (181.6 ± 3.20, 181.91 ± 8.36, and 183.91 ± 2.88 cm for EN, SP, and RE, respectively; *p* = 0.570), and training experience in competitive athletes (*p* = 0.875). Both EN and SP athletes adhered to unified nutrition recommendations made by the dietician of the national team, depending on the training phase of the one-year cycle. The control group followed standard nutritional recommendations for physically active people [29].

All participants were informed about the study aim and protocol and signed their written consent to participate. The project was approved by the Ethics Committee at the Poznan University of Medical Sciences (Poznań, Poland) and conducted following the Helsinki Declaration.

### 4.2. Study Design

The study procedure was adjusted to the annual training plan of EN and SP athletes. All measurements, in both athletes and recreationally-trained participants, were performed in four characteristic training phases as described by Bompa and Buzzichelli [12] (each time within two weeks): (i) first examination in the transition period on October (transition); (ii) second examination in the general subphase of the preparatory phase on December (general); (iii) third examination in the specific subphase of the preparatory phase on February (specific), and (iv) fourth examination in the competition phase on May/June (competition). The training loads performed during each phase of the annual training cycle were presented in our previous research [30]. Two days before the laboratory tests, training volume and intensity were reduced in all participants. All testing procedures were carried out in the Human Movement Laboratory “LaBthletics” of the Poznan University of Physical Education (Poznań, Poland).

### 4.3. Somatic and Physiological Variables

A SECA 285 digital stadiometer (SECA, Hamburg, Germany) was used to measure height and weight. Maximal oxygen uptake (VO_2_max) was measured in the morning using an incremental treadmill test (h/p/cosmos Sports & Medical, Feldschneiderweg, Germany). Initially, the exercise protocol included standing still for three min and walking for three min at a constant speed of 4 km/h. After this introductory part, the speed was increased to 8 km/h and then every 3 min by 2 km/h until the volitional exhaustion was reached. Oxygen consumption was measured with the use of a portable breath-by-breath ergospirometer (MetaMax 3B-R2, MetaSoft Studio software 5.1.0, Cortex Biophysics Gmhb, Leipzig, Germany). VO_2_max was considered achieved if at least three of the following criteria were met: (i) a plateau in VO_2_ despite an increase in running speed, (ii) blood lactate concentration ≥9 mmol/L, (iii) respiratory exchange ratio ≥1.10, and (iv) heart rate ≥ 95% of the age-predicted HR_max_ [31].

### 4.4. Blood Sampling

Venous blood samples were taken three times from an antecubital vein via peripheral venous catheter 1.3 × 32 mm (BD Venflon Pro, Becton Dickinson, Helsingborg, Sweden): (i) before exercise, (ii) immediately after exercise (at exhaustion), and (iii) after 30 min of recovery. Blood samples were taken into two separate tubes with EDTA (2.7 mL) and lithium heparinate (4.9 mL) as anticoagulants (S-monovette, Sarstedt, Nümbrecht, Germany). The first tube was used for the determination of hematocrit (Hct) value and hemoglobin (Hb) concentration. The second tube was used to assess all other compounds: lactate (LA) in whole blood, erythrocyte purine nucleotides (ATP, ADP, AMP, IMP, GTP, guaninediphosphate (GDP), GMP), erythrocyte hypoxanthine-guanine phosphoribosyltransferase (HGPRT) activity, plasma adenosine (Ado), inosine (Ino), guanosine (Guo), and hypoxanthine (Hx).

### 4.5. Hematocrit, Hemoglobin, and Lactate

Hct and Hb measurements were carried out in 10 µL of aspirated blood using the 18-parametric automated hematology analyzer Mythic^®^ 18 (Orphée, Geneva, Switzerland). LA concentration was assayed immediately, before the separation of the erythrocytes from plasma, in whole blood (20 μL) using the spectrophotometric enzymatic method (Biosen C-line, EKF Diagnostics, Barleben, Germany). 

### 4.6. Erythrocyte Isolation

To isolate erythrocytes, the whole blood in the second tube was centrifuged (Universal 320R, Hettich Lab Technology, Tuttlingen, Germany) within 3 min after drawing a sample (1000× *g*, 5 min, 4 °C). Plasma and buffy coat were removed. Then, plasma was divided into aliquots and immediately deep-frozen at −80 °C until the analysis. Erythrocytes were washed three times with buffered 0.9% NaCl solution and centrifuged each time (1000× *g*, 5 min, 4 °C). After the final wash, the resulting erythrocyte pellet was resuspended with a small volume of PBS. The isolated and washed erythrocytes were collected in Modulohm glass capillaries (volume 20 µL, length 75 mm) to obtain hematocrit values (Hct). Hct values were determined in duplicate by the standard microhematocrit method. Next, the samples of washed erythrocytes were deproteinized with an equal volume of 1.3 mol/L HClO4, mixed, and then centrifuged at 16,000× *g* for 5 min at 4 °C. The supernatant (600 μL) was neutralized with 130–160 μL of 3 mol/L K_3_PO_4_ (to pH 5–7). The samples were centrifuged again under the same conditions as previously, and the supernatant was stored at −80 °C until the analysis.

### 4.7. Chromatographic Procedure and Instrumentations

All subsequent measurements in RBC and plasma were performed using high-performance liquid chromatography (HPLC) with UV–VIS detection (Merck-Hitachi/Agilent, Tokio, Japan/Santa Clara, CA, USA) according to the method developed by our group, as described in detail by Smolenski [32] and used by us [3,33]. The system contained high-pressure gradient pump L-6200, 1050 diode array detector, and autosampler AS 2000A with a thermostatic cooler set at 4 °C. An analytical column BDS Hypersil C18, 150 mm × 4.6  mm × 3 μm (Thermo Fisher Scientific, Waltham, MA, USA) protected with guard column 20  mm  ×  4  mm (Phenomenex, Torrance, CA, USA) was used to carry out the separations. Using the ChemStation data system (Agilent, Santa Clara, CA, USA) operating on a PC, all peaks were integrated and quantitative analysis was conducted. Substances were identified by comparing retention times with standards of purines (Sigma Aldrich, St. Louis, Missouri, MA, USA). Mobile phase A consisted of 150 mM potassium phosphate (pH 6.0) and 120 mM potassium chloride in water. Mobile phase B consisted of 15% acetonitrile in buffer A. Gradient elution from 0 to 100% B was completed in 8 min. The System was equilibrated for 5 min before each injection. Calibration was based on external standard injections.

### 4.8. Erythrocyte Purine Nucleotides 

To determine ATP, ADP, AMP, IMP, GTP, GDP, and GMP, 100 μL of supernatant was injected into the sample loop. Purine nucleotides were separated using a gradient elution system at a flow rate of 1 mL/min. Peaks were detected by absorbance at 254 nm. After conversion to Hct, the intra-erythrocyte concentrations of purine nucleotides were expressed as μmol/L RBC. The values of total adenine nucleotide pool (TAN = ATP + ADP + AMP), total guanine nucleotide pool (TGN = GTP + GDP + GMP) were calculated. Adenylate and guanylate energy charge ratios (AEC and GEC, respectively) were calculated using the following formulas:

(1) $$\mathrm{AEC}=\frac{\left[ATP\right]+0.5\left[ADP\right]}{\left[ATP\right]+\left[ADP\right]+\left[AMP\right]}$$

(2) $$\mathrm{GEC}=\frac{\left[GTP\right]+0.5\left[GDP\right]}{\left[GTP\right]+\left[GDP\right]+\left[GMP\right]}$$

### 4.9. Erythrocyte HGPRT Activity 

HGPRT activity was determined in the RBCs lysates. After Hct measurement (standard microhematocrit method), the previously washed erythrocytes were diluted with the Tris buffer (125 mM Tris, pH 7.4) to achieve the Hct value equal to 20%. Erythrocytes prepared in this manner were frozen and thawed twice to increase the lysis of cells. Hemolysates were stored at −80 °C until analysis. To determine the HGPRT activity, 250 μL of the reaction mixture containing 2.5 mmol/L PRPP in 125 mM Tris buffer (pH 7.4), 13 mmol/L MgCl_2_, and 5 mmol/L Hx was prepared. After 5 min of pre-incubation at 37 °C, 25 μL of the previously prepared lysate was added. The samples were incubated for 15 min at 37 °C. Reactions were stopped by adding 0.8 mol/L of HClO_4_ and immediately placed on ice. HGPRT activity was calculated from the IMP concentration increase during 15-min incubation and expressed as nmol IMP/mg Hb/h. Drabkin’s method was used for the determination of hemoglobin concentrations in the erythrocyte lysates.

### 4.10. Plasma Guanosine, Inosine, Adenosine, and Hypoxanthine 

The obtained plasma was deproteinized with 1.3 mol/L perchloric acid (HClO_4_) and once more centrifuged (20,000× *g*, 5 min, 4 °C). An acid supernatant was neutralized with 3 mol/L potassium phosphate K_3_PO_4_, centrifuged (20,000× *g*, 5 min, 4 °C), and stored at −80 °C before analysis. One hundred microliters of supernatant was injected into the sample loop. The concentrations of Guo, Ino, Ado, and Hyp were expressed as μmol/L of plasma.

### 4.11. Statistical Analysis

The sample size was a priori estimated based on the assumption that effect size will be at least medium. Using an α-level of 0.05, a power (1-β) of 0.80, it was calculated that at least eight participants in a single group would be needed to detect a significant change or differences in purine metabolite concentration (RBC and plasma) and erythrocyte HGPRT activity (G*Power software; Heinrich-Heine-Universität, Düsseldorf, Germany). All values are presented as mean ± standard deviation (SD). The Shapiro–Wilk test confirmed the normal distribution of analyzed variables, therefore further analyses were performed using parametric tests. The results obtained during the consecutive phases of the annual training cycle were used to estimate longitudinal changes in the concentration of purine and pyridine nucleotides in red blood cells. For this purpose, the analysis of variance was used. To track the changes over time, the multi-factor analysis of variance (ANOVA/MANOVA) for repeated measures was used. A one-way analysis of variance (ANOVA) was used to determine differences between studied groups. The post hoc Scheffe test was used to determine the significance of the differences within and between consecutive study examinations and between three analyzed groups. The level of significance was set at *p* <0.05. The range of the effect size for differences between measurements, training phases and groups was from small to very large, as follows: purine nucleotides (η^2^ = 0.20–0.99), adenine nucleotides (η^2^ = 0.18–0.97), guanine and pyridine nucleotides (η^2^ = 0.20–0.74), and descriptive and exercise characteristics (η^2^ = 0.24–0.99). The statistical power of ANOVA analyses was 0.63–1.0 for purine nucleotides, 0.58–1.00 for adenine nucleotides, 0.64–0.99 for guanine and pyridine nucleotides and 0.67–1.00 for descriptive and exercise characteristics. All statistical analyses were performed using Statistica 13.3 software (Tibco, Palo Alto, CA, USA). 

## 5. Conclusions

In conclusion, we demonstrated that in competitive athletes an increment in RBC energy status occurred across the annual training period, as assessed by the increments in AEC and ATP/ADP and ADP/AMP ratios. This suggests that the red blood cell metabolism adjusts to the increased physical requirements of the consecutive training phases. Additionally, the increased enzymatic activity (HGPRT) of the purine resynthesis pathway and the accompanying gradual reduction in plasma purine metabolites (Ino, Hx) concentration indicate increased capacity to reduce purine loss and recover erythrocyte purine pool. The concurrent training phase-dependent increment in plasma Ado concentration in athletes supports this mechanism.

## Figures and Tables

**Table 1:** Somatic and exercise characteristics in consecutive training phases (transition, general, specific, competition) in endurance athletes (*n* = 11), sprinters (*n* = 11), and recreational runners (*n* = 11).

Training Phases	Endurance	Sprint	Recreation	ANOVA **
Weight, kg				
transition	74.2 ± 3.28 ^†c^	80.6 ± 4.80 ^gsc^	78.4 ± 3.50 ^gsc^	0.002
general	73.6 ± 3.07 ^†‡c^	79.1 ± 4.80	79.3 ± 3.44	0.002
specific	73.6 ± 3.11 ^†‡c^	79.6 ± 4.80	79.1 ± 3.18	0.001
competition	72.6 ± 3.78 ^†‡^	79.2 ± 4.33	79.0 ± 3.00	0.000
ANOVA *	0.000	0.000	0.001	
BMI, kg/m^2^				
transition	22.5 ± 1.46 ^†c^	24.5 ± 1.79 ^gsc^	23.2 ± 0.80	0.010
general	22.4 ± 1.33 ^†c^	24.0 ± 1.76	23.4 ± 0.77	0.025
specific	22.3 ± 1.35 ^†c^	24.2 ± 1.77	23.4 ± 0.76	0.014
competition	22.0 ± 1.51 ^†^	24.0 ± 1.80	23.6 ± 0.9	0.008
ANOVA *	0.000	0.000	0.067	
Hb, g/dl				
transition	16.1 ± 0.54 ^†‡^	15.4 ± 0.48	15.1 ± 0.36	0.000
general	16.0 ± 0.93 ^‡^	15.5 ± 0.91	14.9 ± 0.30	0.005
specific	16.1 ± 0.68 ^‡^	15.5 ± 0.86	15.0 ± 0.43	0.005
competition	16.2 ± 0.92 ^†‡^	15.3 ± 0.72	15.1 ± 0.34	0.002
ANOVA *	0.819	0.615	0.040	
Hct, %				
transition	47.1 ± 2.13 ^†‡^	44.2 ± 0.92 ^gs^	43.3 ± 0.57	0.000
general	47.0 ± 2.07 ^†‡^	43.9 ± 0.91 ^c^	43.2 ± 0.86	0.000
specific	47.0 ± 2.17 ^†‡^	43.9 ± 0.91	43.6 ± 0.83	0.000
competition	47.2 ± 3.34 ^†‡^	44.4 ± 0.93	43.5 ± 0.81	0.000
ANOVA *	0.982	0.000	0.438	
LA rest, mmol/L				
transition	1.01 ± 0.25	0.95 ± 0.18	1.00 ± 0.15	0.731
general	1.28 ± 0.32	1.08 ± 0.21 ^s^	1.01 ± 0.19	0.042
specific	1.18 ± 0.49	0.87 ± 0.20 ^c^	1.06 ± 0.12	0.081
competition	1.38 ± 0.49	1.07 ± 0.19	1.05 ± 0.09	0.031
ANOVA *	0.171	0.001	0.649	
LA max, mmol/L				
transition	10.01 ± 0.93	10.02 ± 0.93	10.31 ± 0.88	0.677
general	10.73 ± 1.16	10.21 ± 1.09	10.13 ± 1.22	0.436
specific	10.25 ± 0.90	10.68 ± 0.60	10.18 ± 1.03	0.347
competition	10.40 ± 1.17	10.13 ± 0.74	10.22 ± 1.11	0.819
ANOVA *	0.119	0.249	0.855	
VO_2max_, mL·kg^−1^·min^−1^				
transition	65.5 ± 5.34 ^†‡sc^	52.6 ± 3.89 ^gs^	48.1 ± 3.24	0.000
general	67.4 ± 6.09 ^†‡^	55.5 ± 3.59 ^‡^	47.4 ± 2.94	0.000
specific	68.9 ± 6.15 ^†‡^	56.3 ± 3.24 ^‡^	47.7 ± 2.87	0.000
competition	68.7 ± 6.67 ^†‡^	54.7 ± 2.56 ^‡^	47.9 ± 2.88	0.000
ANOVA *	0.000	0.001	0.195	

Values are given as means ± SD. * one-way ANOVA between terms within one group. ** one-way ANOVA between groups within one training phase. † significantly different from sprinters; ‡ significantly different from recreational runners. g significantly different from general; s significantly different from specific; c significantly different from the competition training phase. BMI: body mass index; Hb: hemoglobin; Hct: hematocrit; LA: lactate; VO_2_max: maximal oxygen uptake.

## Supplementary Materials

The following are available online at https://www.mdpi.com/2218-1989/10/1/5/s1, Table S1. Red blood cell guanine nucleotides (GTP, GDP, GMP) concentration, total guanine nucleotide (TGN), guanylate energy charge (GEC), at rest, post-exercise, and after 30 min of recovery in consecutive training phases (transition, general, specific, competition) in endurance athletes (*n* = 11), sprinters (*n* = 11), and recreational runners (*n* = 11).
