# Supplementary material for: The Effect of Training on Erythrocyte Energy Status and Plasma Purine Metabolites in Athletes
Source: Metabolites. 2019 Dec 19;10(1):5. doi: 10.3390/metabo10010005 (PMC7022963; doi:10.3390/metabo10010005)
Supplement: Supplementary file 1 [file metabolites-10-00005-s001.pdf]

Supplementary File

Table S1. Red blood cell guanine nucleotides (GTP, GDP, GMP) concentration, total guanine nucleotide (TGN), guanylate energy charge (GEC), at rest, post-exercise and after 30 minutes of recovery in consecutive training phases (transition, general, specific, competition) in endurance athletes (*n* = 11), sprinters (*n* = 11), and recreational runners (*n* = 11).

training phases	ENDURANCE			SPRINT			RECREATION		
	Rest	Post-exercise	30' recovery	Rest	Post-exercise	30' recovery	Rest	Post-exercise	30' recovery
GTP, mmol/L RBC									
Transition	80.9 ± 5.23	80.7 ± 5.09	80.4 ± 4.73	85.7 ± 5.94	85.5 ± 5.65	85.1 ± 5.87	78.3 ± 5.99	77.9 ± 6.09	77.6 ± 6.58
General	81.3 ± 4.75	80.4 ± 5.12	80.3± 4.90	85.0 ± 6.2	84.6 ± 5.91	84.4 ± 6.14	78.4 ± 5.48	78.1 ± 5.35	78.0 ± 5.40
Specific	81.1 ± 4.16	80.9 ± 4.8	80.5 ± 4.1	85.6 ± 6.41	85.6 ± 6.13	85.0 ± 6.52	78.1 ± 5.99	77.6 ± 6.09	77.4 ± 6.58
Competition	81.2 ± 4.35	80.6 ± 4.37	80.3 ± 4.43	85.5 ± 6.29	85.2 ± 5.64	84.9 ± 5.95	78.214 ± 5.56	77.8 ± 5.86	77.7 ± 6.33
GDP, mmol/L RBC									
Transition	22.9 ± 3.49	23.2 ± 3.42	23.1 ± 3.60	25.2 ± 4.25†	25.5 ± 4.55	25.4 ± 4.3	19.4 ± 4.63	19.7 ± 4.76	19.5 ± 4.75
General	22.7 ± 3.42	22.9 ± 3.63	22.9 ± 3.57	25.9 ± 4.6	26.0 ± 4.57	25.9 ± 4.54	19.7 ± 4.77	20.0 ± 4.82	19.8 ± 4.80
Specific	23.0 ± 3.55	23.2 ± 3.43	23.1 ± 3.53	25.7 ± 4.84	25.8 ± 4.76	25.6 ± 4.79	19.7 ± 4.77	20.0 ± 4.82	19.8 ± 4.8
Competition	23.3 ± 3.44	23.4 ± 3.33	23.4 ± 3.46	25.9 ± 4.53	26.0 ± 4.48	25.9 ± 4.5	19.5 ± 5.1	19.7 ± 5.38	19.6 ± 5.27
GMP, mmol/L RBC									
Transition	20.7 ± 3.75	20.5 ± 3.92	20.6 ± 3.77	22.8 ± 3.57 ‡	22.5 ± 3.59	22.5 ± 3.46	17.1 ± 4.49	16.8 ± 4.45	17.0 ± 4.66
General	20.4 ± 3.34	20.0 ± 3.11	20.5 ± 3.14	22.6± 3.58	22.2 ± 3.82	22.4 ± 3.43	16.9 ± 4.41	16.5 ± 4.58	16.8 ± 4.63
Specific	20.6 ± 3.02	20.2 ± 2.91	20.6 ± 2.91	23.0 ± 3.08	22.6 ± 3.08	22.7 ± 2.98	16.6 ± 5.15	16.3 ± 5.1	16.5 ± 5.35
Competition	20.8 ± 2.88	20.5 ± 2.72	20.7 ± 2.88	22.7 ± 3.16	22.5 ± 3.17	22.6 ± 3.21	16.9 ± 5.03	16.5 ± 4.74	16.6 ± 5.13
TGN, mmol/L RBC									
Transition	124.5 ± 11.51	124.3 ± 11.27	124.1 ± 10.70	133.6 ± 13.01 ‡	133.6 ± 12.98 ‡	133.0 ± 12.92	114.7 ± 14.82	114.3 ± 15.01	114.1 ± 15.61
General	124.4 ± 10.43	123.3 ± 10.61	123.6 ± 10.25	133.4 ± 13.53	132.8 ± 13.47	132.6 ± 13.22	114.9 ± 14.15	114.6 ± 14.24	114.5 ± 14.32
Specific	124.7 ± 9.26	124.3 ± 9.9	124.2 ± 9.29	134.4 ± 13.55 ‡	133.9 ± 13.29	133.4 ± 13.32	114.0 ± 15.24	114.1 ± 15.48	113.8 ± 16.09
Competition	125.3 ± 9.87	124.4 ± 9.56	124.4 ± 10.14	134.0 ± 13.35	133.7 ± 12.65	133.3 ± 13.02	114.3 ± 15.0	114.3 ± 15.87	113.9 ± 16.33
GEC									
Transition	0.74 ± 0.023	0.74 ± 0.025	0.74 ± 0.025	0.74 ± 0.018	0.74 ± 0.019	0.74 ± 0.018	0.77 ± 0.03	0.77 ± 0.03	0.77 ± 0.03

General	0.75 ± 0.021	0.75 ± 0.019	0.74 ± 0.020	0.74 ± 0.018	0.74 ± 0.02	0.74 ± 0.018	0.77 ± 0.032	0.77 ± 0.034	0.77 ± 0.034
Specific	0.74 ± 0.022	0.75 ± 0.02	0.74 ± 0.021	0.73 ± 0.014	0.74 ± 0.014	0.73 ± 0.014	0.77 ± 0.036	0.77 ± 0.036	0.77 ± 0.037
Competition	0.74 ± 0.018	0.74 ± 0.017	0.74 ± 0.017	0.74 ± 0.015	0.74 ± 0.015	0.74 ± 0.01	0.77 ± 0.036	0.77 ± 0.035	0.77 ± 0.036

Values are given as means ± SD. † significantly different from Post-exercise ($p \leq 0.05$); ‡ significantly different from 30'recovery ($p \leq 0.05$). GDP - guanosine-5′- diphosphate. GEC - guanylate energy charge; was evaluated according to the formula by Atkinson GEC = ([GTP] + 0.5[GDP]) / ([GTP] + [GDP] + [GMP]). GMP - guanosine-5′- monophosphate. GTP - guanosine-5′- triphosphate. TGN - total guanine nucleotides = [GTP] + [GDP] + [GMP].
